# Sleep deficits but no metabolic deficits in premanifest Huntington's disease

**DOI:** 10.1002/ana.24495

**Published:** 2015-08-21

**Authors:** Alpar S. Lazar, Francesca Panin, Anna O. G. Goodman, Stanley E. Lazic, Zsolt I. Lazar, Sarah L. Mason, Lorraine Rogers, Peter R. Murgatroyd, Laura P. E. Watson, Priya Singh, Beth Borowsky, John M. Shneerson, Roger A. Barker

**Affiliations:** ^1^ John van Geest Centre for Brain RepairDepartment of Clinical NeurosciencesUniversity of CambridgeCambridgeUnited Kingdom; ^2^ Faculty of Medical ScienceAnglia Ruskin UniversityCambridgeUnited Kingdom; ^3^ In Silico Lead Discovery, Novartis Institutes for Biomedical ResearchBaselSwitzerland; ^4^ Department of PhysicsBabes‐Bolyai UniversityCluj‐NapocaRomania; ^5^ Respiratory Support and Sleep Centre, Papworth HospitalCambridgeUnited Kingdom; ^6^ National Institute for Health Research/Wellcome Trust Clinical Research Facility, Addenbrooke's HospitalCambridgeUnited Kingdom; ^7^ University of Cambridge Metabolic Research Laboratories, Wellcome Trust–Medical Research Council Institute of Metabolic Science, Addenbrookes HospitalCambridgeUnited Kingdom; ^8^ Medical Research Council Human Nutrition Research, Elsie Widdowson LaboratoryCambridgeUnited Kingdom; ^9^ CHDI Management/CHDI FoundationPrincetonNJUnited States of America

## Abstract

**Objective:**

Huntington disease (HD) is a fatal autosomal dominant, neurodegenerative condition characterized by progressively worsening motor and nonmotor problems including cognitive and neuropsychiatric disturbances, along with sleep abnormalities and weight loss. However, it is not known whether sleep disturbances and metabolic abnormalities underlying the weight loss are present at a premanifest stage.

**Methods:**

We performed a comprehensive sleep and metabolic study in 38 premanifest gene carrier individuals and 36 age‐ and sex‐matched controls. The study consisted of 2 weeks of actigraphy at home, 2 nights of polysomnography and multiple sleep latency tests in the laboratory, and body composition assessment using dual energy x‐ray absorptiometry scanning with energy expenditure measured over 10 days at home by doubly labeled water and for 36 hours in the laboratory by indirect calorimetry along with detailed cognitive and clinical assessments. We performed a principal component analyses across all measures within each studied domain.

**Results:**

Compared to controls, premanifest gene carriers had more disrupted sleep, which was best characterized by a fragmented sleep profile. These abnormalities, as well as a theta power (4–7Hz) decrease in rapid eye movement sleep, were associated with disease burden score. Objectively measured sleep problems coincided with the development of cognitive, affective, and subtle motor deficits and were not associated with any metabolic alterations.

**Interpretation:**

The results show that among the earliest abnormalities in premanifest HD is sleep disturbances. This raises questions as to where the pathology in HD begins and also whether it could drive some of the early features and even possibly the pathology. Ann Neurol 2015;78:630–648

Huntington disease (HD) is a fatal autosomal dominant neurodegenerative condition that affects approximately 14 to 16 individuals per 100,000 and typically presents in midlife.^1^, ^2^ It is caused by an abnormal expansion of a trinucleotide cytosine–adenosine–guanosine repeat (CAG)^3^ in exon 1 of the huntingtin gene leading to the ubiquitous expression of mutant huntingtin (*Htt*).^4^ It is characterized by progressively worsening motor and nonmotor deficits including cognitive abnormalities (which over time lead to dementia), neuropsychiatric symptoms,^5^ weight loss,^6^ and sleep and circadian disturbances (for a review see Videnovic et al^7^). Although pathogenic pathways are beginning to be unraveled offering targets for treatment,^8^ the precise pathophysiological mechanisms of HD are poorly understood.^9^ Previously we have shown that sleep disturbances are present in early manifest disease,^10^ and several studies on transgenic animal models of HD have shown that sleep progressively worsens as the disease develops^11^, ^12^, ^13^, ^14^ dependently on age and gene dosage.^15^ However, we do not know whether sleep problems are present during the premanifest stage in individuals carrying the HD gene mutation. If so, it may be a useful predictor of the onset of clinical features in gene carriers, and may contribute to some of the early clinical features of the condition, including the well‐described cognitive deficits.^16^

Similarly, altered energy expenditure and balance have been shown in HD patients,^17^, ^18^, ^19^ and might underlie the progressive weight loss reported to occur early on in this condition.^6^, ^20^ This raises questions as to whether metabolic problems in HD also exist ahead of motor features and disease diagnosis and may occur at the same time as sleep and circadian problems, suggesting a common neuropathological substrate that may involve the hypothalamus.^21^

In the present study, we aimed to answer these questions by undertaking a comprehensive metabolic and sleep investigation both in the field (doubly labeled water [DLW], actigraphy, sleep diaries) and in the laboratory (indirect calorimetry, polysomnography [PSG], and multiple sleep latency tests [MSLTs]) in a large cohort of premanifest HD (Pre‐HD) gene carriers and age‐ and sex‐matched controls. We hypothesized that sleep and metabolic disturbances would: (1) be present in the premanifest stage, (2) relate to estimated disease burden, and (3) coincide with early alterations in cognition.

## Subjects and Methods

### Participants

All aspects of the study were approved by the local research ethics committees and conformed to the Declaration of Helsinki and International Conference on Harmonization–Good Clinical Practice. After written informed consent was obtained, 38 premanifest participants with a known HD gene mutation and 36 sex‐, age‐, and ethnically balanced control participants took part in the metabolic and/or sleep studies (Table 1). Separate informed consent was required for each study. The cognitive assessments and metabolic studies were performed at the Huntington's Disease Research Clinic at the John van Geest Centre for Brain Repair and the Metabolic Research Area within the National Institute for Health Research/Wellcome Trust Cambridge Clinical Research Facility, Cambridge, United Kingdom. The sleep studies were conducted in the sleep unit at the Respiratory Support and Sleep Centre, Papworth Hospital, United Kingdom. Altogether 30 patients and 20 controls participated in all 3 studies, with a median time difference of 5 months (interquartile range = 12 months) between the studies. In all cases the sleep study preceded the metabolic studies.

**Table 1:** Demographic Description of Patient Groups

				Group Differences		
Demographic Data	Controls, n = 36, Mean (SD)	prHD, n = 38, Mean (SD)	eHD, n = 8, Mean (SD)	Ctrl–prHD	Ctrl–eHD	prHD–eHD
Sex, No.	18 M, 18 F	13 M, 25 F	4 M, 4 F	0.19	1.0	0.4
Age, yr	44.2 (15)	43.0 (11.2)	56.3 (7.2)	0.99	0.015^a^	0.002^a^
BMI, kg/m^2^	24.6 (3.0)	26.0 (5.0)	21.5 (2.2)	0.35	0.01^a^	0.005^a^
CAGn	N/A	41.6 (2.4)	42 (1.7)	N/A	N/A	0.43
Disease burden score	N/A	247.2 (65.5)	367.3 (78.9)	N/A	N/A	0.0008^a^
UHDRS global score	N/A	1.6 (2.1)	19.3 (7.1)	N/A	N/A	<0.0001^a^
Total functional capacity	N/A	12.6 (0.9)	9.8 (1.8)	N/A	N/A	<0.0001^a^
Independence	N/A	98.2 (5.0)	81.7 (7.5)	N/A	N/A	<0.001^a^

Group differences are analyzed by Mann–Whitney *U* test. Age is reported at the time subjects completed their first study in this project. Disease burden score was calculated using the published formula: (CAGn − 35.5) × age (see Subjects and Methods).

a Significant effect.

eHD = early Huntington disease; Ctrl = control; prHD = premanifest Huntington disease; CAGn = number of cytosine–adenosine–guanosine repeats; BMI = body mass index; UHDRS = Unified Huntington's Disease Rating Scale; SD = standard deviation; M = male; F = female; N/A = not applicable.

Inclusion/exclusion criteria for the premanifest group were: (1) a CAG repeat length ≥ 39; (2) not clinically diagnosed as having manifest HD as indicated by a diagnostic confidence score < 2 on the Unified Huntington's Disease Rating Scale (UHDRS); (3) no known metabolic, endocrine, or sleep disorder; and (4) a nonsmoking/light smoking status. Inclusion/exclusion criteria for the control group were: (1) no family history of HD or other known neurological, endocrine, or sleep disorder; (2) no ongoing medical or psychiatric condition; (3) nonsmoking/light smoking status; and (4) an age, sex, and body mass index (BMI) match to a Pre‐HD participant. Of the included participants, 12 pre‐HD patients and 6 controls were taking medications that could have had a possible effect on sleep and/or metabolism, such as antidepressants. One premanifest participant was excluded from the metabolic studies due to diabetes. A disease burden score (DBS) was calculated for all premanifest participants according to a standard formula: (CAGn − 35.5) × age, where CAGn = number of CAG repeats.^22^ The studied groups (control and Pre‐HD) did not significantly differ in terms of age, BMI, and sex (see Table 1). For comparison with the premanifest group, we included sleep data from a previously studied (and published) cohort of 8 early manifest patients^10^ while also undertaking further in‐depth sleep analyses of this previously collected data. These patients were significantly older and had a lower BMI than those in the current study. They also had as expected a higher DBS and worse scores across the basic clinical measures as compared to premanifest patients.

### Procedures

Baseline clinical, cognitive, and psychiatric assessments consisted of:

A. The motor and functional sections of the UHDRS.^23^
B. A selection of standardized tests assessing (1) global cognitive performance: Montreal Cognitive Assessment (MoCA)^24^; (2) learning and verbal memory: Hopkins Verbal Learning Test–Revised (HVLT‐R)^25^; (3) executive function: the Verbal Fluency Test^26^ and Trail Making Tests B^27^; (4) psychomotor speed: Trail Making Tests A^27^ and Symbol Digit Test^28^; (5) motor skills: the Hand Tapping Test^29^; (6) olfactory perception: the Olfactory Discrimination and Identification Test^30^; (7) affect: the Montgomery–Åsberg Depression Rating Scale (MADRS),^31^ the Beck Depression Inventory II (BDI‐II),^32^ and the Apathy Evaluation Scale^33^; and (8) impulsiveness: Barratt Impulsiveness Scale^34^ (Table 2).

**Table 2:** Neuropsychological Profiles and Motor Performance

	Controls			Pre‐HD				
Studied Measures	No.	Estimate	95% CI	No.	Estimate	95% CI	Genotype Effect, *p* (Cohen *f* ^*2*^)	DBS Effect, *p*
Global cognitive performance								
MoCA score, max = 30	24	25.7	25.1–26.3	34	24.5	23.9–25.1	0.008 (0.14)^a^	0.0006^a^
Executive functions								
Semantic fluency^b^	26	22.2	20.0–24.4	33	21.6	19.6–23.7	0.698	0.4346
Phonemic fluency^b^	26	43.4	38.1–49.4	34	37.2	33.1–41.9	0.086	0.292
Trail Making B, s	25	49.4	42.3–57.7	35	56.1	49.0–64.3	0.222	0.187
Verbal memory								
HVLT‐R average recall,^b^ max = 12	26	9.0	8.4–9.7	34	7.9	7.3–8.5	0.013 (0.12)^a^	0.0417^a^
Olfactory perception								
Discrimination,^b^ max = 16	24	12.7	11.9–13.5	32	11.8	11.1–12.5	0.093	0.003^a^
Identification,^b^ max = 16	25	12.8	12.0–13.6	32	11.9	11.2–12.7	0.117	0.2363
Psychomotor speed								
Trail Making A, s	25	28.5	25.2–32.2	35	30.1	27.1–33.5	0.493	0.8843
Symbol digit^b^	12	49.6	43.7–55.6	27	49.6	45.7–53.5	0.997	0.0919
Motor function								
Right hand taps^b^	23	119.9	110.8–128.9	34	111.7	103.9–119.5	0.183	0.6962
Left hand taps^b^	23	110.3	103.1–117.5	34	100.0	93.8–106.1	0.036 (0.09)^a^	0.3059
Depression and apathy								
MADRS, max = 60	24	1.5	0.2–3.1	33	3.1	1.7–5.0	0.135	0.7651
BDI‐II, max = 63	25	2.1	1.1–3.6	31	3.7	2.3–5.8	0.122	0.4927
AES, max = 72	23	25.1	23.1–27.2	29	24.3	22.5–26.1	0.556	0.4452
Impulsivity								
Barratts, max = 120	20	3.3	3.2–3.3	28	3.2	3.2–3.3	0.907	0.3033

Number of participants in a category, estimates (least squares means) and 95% CIs are indicated for each group adjusted for age and sex. For each measure, the genotype effect (controls vs Pre‐HD) and the DBS is indicated. Genotype is performed by mixed model analysis of variance controlled for age and sex. Effects of age and sex are not indicated. Effect of DBS is analyzed within the Pre‐HD group using a multivariate regression including DBS and sex. Effect size is indicated only for significant effects.

a Significant effects.

b Total correct.

AES = Apathy Evaluation Scale; BDI‐II = Beck Depression Inventory II; CI = confidence interval; DBS = disease burden score; HVLT‐R = Hopkins Verbal Learning Test–Revised; MADRS = Montgomery–Åsberg Depression Rating Scale; MoCA = Montreal Cognitive Assessment; Pre‐HD = premanifest Huntington disease.

The sleep study consisted of:

A. Validated sleep questionnaires to assess (1) diurnal preference: the Morningness‐Eveningness Questionnaire (MEQ)^35^; (2) subjective sleep quality: the Pittsburgh Sleep Quality Index (PSQI)^36^ and the Functional Outcomes of Sleep Questionnaire^37^; (3) daytime sleepiness: the Epworth Sleepiness Scale^38^; (4) habitual self‐reported sleep–wake timing: PSQI; (5) preferred sleep–wake timing: MEQ; and (6) timing of evening tiredness sufficient to go to sleep: MEQ.
B. A 2‐week‐long field/home study to assess sleep–wake timing as measured by actigraphy in the habitual environment. Participants wore Actiwatches (Cambridge Neurotechnology, Cambridge, UK) on the nondominant wrist for 14 consecutive days preceding the PSG study. Actograms were analyzed according to a previously published algorithm.^10^
C. An inpatient laboratory‐based study consisting of 2 consecutive nights of PSG to assess objective sleep quality and sleepiness using MSLTs following a previously described methodology.^10^ A full clinical electroencephalographic (EEG)‐PSG setup was used, and PSG data were recorded on an Embla S7000 (Embla Systems, Ontario, Canada). The EEG was recorded using C3 and C4 derivations and reference electrodes placed at the mastoid area (A1 and A2) with a common reference electrode placed at Pz. The second (study) night was used for the actual analysis. Sleep staging was performed in 30‐second epochs according to standard criteria (Rechtschaffen and Kales criteria) by scorers blind to the identity of patients. We analyzed multiple objective sleep parameters (Table 3). Breathing events were scored using standard criteria according to the American Academy of Sleep.^39^ EEG data were stored at 200Hz. The low‐pass filter was set at 70Hz, and the high‐pass filter was set at 0.3Hz.
D. Spectral analyses. The active EEG derivations were rereferenced offline to the mastoid derivation (A1 and A2) from the contralateral hemisphere. All EEG artifacts (eg, muscle activity/sweating) for each individual EEG channel were visually identified by an experienced scorer and annotated on a 3‐second basis using the EEG browser software Vitascore version 1.5. Thereafter, all EEG channels were exported and raw EEG data further analyzed. We applied algorithms based on the NumPy, SciPy, and Matplotlib libraries for scientific computing^40^, ^41^ to extract EEG activity–related measures. Analysis was limited to data recorded between lights out and lights on times. The 2 sleep stage sets—(1) rapid eye movement (REM) and (2) non‐REM (NREM) 1, 2, 3, and 4—were analyzed independently. EEG activity was calculated as the power spectral density averaged over the whole night. Artifact‐free segments belonging to the sleep stages of interest were concatenated and power values were calculated as averages over detrended Hanning windowed 1,024 bins, that is, 5.12‐second long epochs with 50% overlap using the Welch's periodogram method implemented in the Matplotlib package. In the first stage, the obtained spectral density was coarse grained by calculating averages over subsequent 1Hz‐wide bins between 0.5 and 40.5Hz. To eliminate the discontinuous dependence of the averages on band boundaries, the averaging was performed by numerically integrating over the different regions of the cubic spline interpolated spectrum.^42^ The spectral values in each frequency bin were normalized to the total power of the studied EEG spectra 0.5 and 40.5Hz. The returned relative spectral values, *x*, were logit transformed according to published recommendations following the formula ‘*y* = log[*x*/(1−*x*)]’.^43^

**Table 3:** Sleep Parameters as Measured by Polysomnography

	Control, n = 25		prHD, n = 31		eHD, n = 8			Contrasts, *p*			
Studied Measures	Estimate	95% CI	Estimate	95% CI	Estimate	95% CI	Group Main Effects, *p* (Cohen *f* ^*2*^)	Ctrl–prHD	Ctrl–eHD	prHD–eHD	DBS Effect, *p*
Sleep timing in the lab											
Bedtime, hh:mm	22:55	22:37–23:13	22:41	22:24–22:57	22:02	21:28–22:35	0.026 (0.13)^a^	0.237	0.007^a^	0.043^a^	0.015^a^
Wakeup time, hh:mm	07:05	06:57–07:12	07:08	07:01–07:14	07:10	06:55–07:21	0.741	0.537	0.514	0.792	0.610
Sleep onset time, hh:mm	23:11	22:51–23:31	22:53	22:35–23:11	22:28	21:54–23:04	0.108	0.191	0.045^a^	0.223	0.073
General sleep parameters											
TST, min	420.0	393.8–446.1	415.3	391.4–439.3	396.0	347.9–444.2	0.688	0.794	0.390	0.478	0.602
TIB, min	491.3	472.3–510.2	510.2	492.9–527.6	562.4	527.5–597.4	0.003 (0.21)^a^	0.144	0.0008^a^	0.010^a^	0.004^a^
SE	86.4	83.5–89.1	83.6	80.8–86.3	74.7	66.7–81.2	0.005 (0.20)^a^	0.167	0.001^a^	0.013^a^	0.024^a^
WASO, min	37.3	29.9–46.4	55.8	45.7–68.1	94.6	63.5–140.5	0.0003 (0.31)^a^	0.008^a^	0.0001^a^	0.022^a^	0.008^a^
SPT, min	471.5	449.4–492.4	493.8	474.8–512.0	519.8	483.2–553.6	0.061	0.118	0.027^a^	0.206	0.060
SL, min	11.5	8.0–16.3	9.6	6.9–13.3	17.2	9.1–32.1	0.266	0.457	0.273	0.111	0.166
LPS, min	14.8	9.9–21.8	17.9	12.5–25.5	31.1	15.3–62.2	0.197	0.468	0.073	0.170	0.229
REM latency, min	59.0	47.2–73.7	75.1	61.3–91.9	139.2	92.8–208.7	0.002 (0.23)^a^	0.114	0.0005^a^	0.009^a^	0.350
SWS latency, min	21.7	15.3–30.6	29.7	21.8–40.3	23.2	12.3–42.8	0.378	0.176	0.856	0.478	0.156
Sleep structure											
Stage 1, % of TST	10.3	8.5–12.1	11.2	9.6–12.8	16.4	13.1–19.6	0.007 (0.18)^a^	0.449	0.002^a^	0.006^a^	0.071
Stage 2, % of TST	54.2	51.1–57.4	53.5	50.6–56.4	51.0	45.2–56.8	0.627	0.735	0.336	0.444	0.485
SWS, % of TST	13.2	10.0–16.3	14.9	12.0–17.8	17.3	11.5–23.1	0.431	0.412	0.221	0.472	0.806
REM, % of TST	22.3	19.9–24.7	20.4	18.1–22.6	15.3	10.9–19.8	0.030 (0.13)^a^	0.238	0.0085^a^	0.050^a^	0.079
Sleep continuity											
Arousals^b^	11.1	9.6–12.7	13.9	12.3–15.8	18.4	14.3–23.5	0.002 (0.24)^a^	0.017^a^	0.0009^a^	0.053	0.020^a^
Sleep bout length, min^c^	14.0	11.7–16.8	10.0	8.6–11.6	7.6	5.8–10.2	0.001 (0.26)^a^	0.005^a^	0.001^a^	0.104	0.031^a^
REM bout length, min^d^	5.6	4.7–6.7	4.0	3.3–4.7	3.7	2.5–5.2	0.016 (0.15)^a^	0.009^a^	0.037^a^	0.684	0.085
Sleep stage changes^e^	23.0	20.8–25.3	26.4	24.1–28.9	31.7	26.5–37.9	0.006 (0.19)^a^	0.039^a^	0.003^a^	0.073	0.078
Fast sleep stage changes^f^	1.7	1.3–2.1	2.9	2.4–3.5	4.0	2.8–5.6	0.0002 (0.33)^a^	0.0010^a^	0.0004^a^	0.116	0.040^a^
AHI^b^	3.3	2.1–5.0	4.1	2.7–5.9	3.3	1.3–7.0	0.755	0.482	0.989	0.656	0.523
Objective sleepiness											
Sleep latency (MSLT), min	11.2	10.0–12.5	9.2	8.3–10.2	17.3	14.2–21.1	<0.0001 (0.54)^a^	0.011^a^	0.0004^a^	<0.0001^a^	0.005^a^

Estimates (least squares means) and 95% CIs are indicated for each group adjusted for age and sex. For each measure, the group main effect and the disease burden effect (DBS) are indicated. Group comparisons involved a mixed model analysis of variance controlled for age and sex. Effects of age and sex are not indicated. Effect of DBS is analyzed within the entire prHD and eHD groups using a multivariate linear regression model including the predictors DBS and sex. Effect size is indicated only for significant effects. For detailed description of the measures please refer to the Subjects and Methods section.

a Significant effects.

b Events per hour of TIB (shift to stage 1, wake, or movement).

c Sleep bout length = TST/number of awakenings.

d REM bout length = REM/number of entries into REM.

e Events (shifts between stages) per hour of TST.

f Fast sleep stage changes = periods of 1.5 minutes with 3 different 30‐second‐long sleep stages per hour of sleep.

AHI = Apnea–hypopnea index (apnea and hypopnea events/hour of TIB); CI = confidence interval; Ctrl = controls; DBS = disease burden score; eHD = early Huntington disease; hh:mm = hours:minutes; LPS = latency to persistent sleep (10 minutes of continuous sleep); MSLT = Multiple Sleep Latency Test (averaged sleep latency values are indicated across the 5 sleep opportunities); prHD = premanifest Huntington disease; REM = rapid eye movement sleep; SE = sleep efficiency (TST/TIB %); SL = sleep latency; SPT = sleep period time (time between sleep onset and the final awakening); SWS = slow wave sleep (stages 3 and 4); TIB = time in bed; TST = total sleep time; WASO = wake after sleep onset (total wake time between the sleep onset and the final awakening); REM latency = time between sleep onset and the first REM epoch; SWS = time between sleep onset and the first epoch of SWS.

The metabolic study consisted of:

A. Dual energy x‐ray absorptiometry to assess body composition followed by 36 hours of whole body indirect calorimetry to assess energy expenditure in the laboratory according to published methodology.^19^ Blood samples for testosterone, cortisol, vitamin D, and leptin were taken at 9 am after the second night sleep, and after a minimum 10‐hour fast and the basal metabolic rate assessment, which all occurred before the participant got out of bed. Subjectively perceived hunger and satiety were assessed by a visual analogue scale before and after meals.^44^
B. A 10‐day free living measurement of total energy expenditure (TEE) using DLW according to a standard methodology.^45^ Average daily activity–related energy expenditure (AEE; kJ/day/kg) was calculated as the average 24‐hour TEE measured by DLW, minus the basal metabolic rate (BMR) measured from the indirect calorimetry, whereas the physical activity level (PAL) was the ratio of these same variables (TEE/BMR). Finally, the variance of physical activity (VPA) between the laboratory and the field condition was calculated by subtracting the 24‐hour TEE measured by indirect calorimetry from the 24‐hour TEE as measured by DLW, according to the study performed by Pratley et al.^18^

### Statistical Analyses

We did not define a primary outcome in this exploratory study of Pre‐HD patients because abnormalities on any of the measured variables are noteworthy and relevant. This posed a problem of limiting the number of false positives while simultaneously retaining sufficient power to detect differences between patients and controls, which a priori are expected to be small as the patients do not yet have manifest disease. A principal component analysis (PCA) was therefore used to reduce sets of (often highly correlated) measurements to smaller sets of uncorrelated variables. This allowed us to create a new set of variables (the principal components [PCs]), which involved taking a linear combination of the original variables. One of the main features of PCA is that the first principal component (PC1) accounts for most of the variation in the data, and each subsequent PC accounts for less and less of the remaining variation. Thus by analyzing the first few PCs rather than the original variables, we were able to limit the number of statistical tests while still capturing the major effects that are present. A PCA was conducted for each of the 4 studied domains of measurements (1 PCA for the data in each of Tables 2, 3, 4, 5). The first PC had the highest association with disease and was therefore the only one examined, with the exception of the metabolic data, where PC1 was dominated by sex differences and therefore PC2 was analyzed. A priori, only PCs with large eigenvalues (>1.5) were examined, which limited the analysis to the first 2 PCs.

**Table 4:** Diurnal Preference, Habitual Sleep–Wake Timing, Subjective Sleep Quality, and Sleepiness

	Control, n = 25		Pre‐HD, n = 31			
Studied Measures	Estimate	95% CI	Estimate	95% CI	Group Main Effects, *p*	DBS Effect, *p*
Diurnal preference (MEQ), range = 16–86^d^	57.0	53.3–60.7	56.3	52.9–59.6	0.761	0.120
Preferred sleep–wake timing (MEQ), hh:mm						
Bedtime^a^	23:03	22:39–23:28	23:00	22:39–23:22	0.855	0.133
Get‐up time^a^	07:49	07:27–08:11	07:56	07:36–08:16	0.618	0.969
Time in bed^b^	08:45	08:23–09:07	08:56	08:36–09:16	0.490	0.043^c^
Habitual self‐reported sleep–wake timing (PSQI), hh:mm						
Bedtime^a^	23:11	22:45–23:38	22:55	22:30–23:20	0.381	0.163
Get‐up time^a^	07:15	06:48–07:41	07:31	07:06–07:55	0.390	0.190
Sleep latency, min	15.2	11.4–20.1	19.7	15.2–25.4	0.184	0.150
Time in bed^b^	08:04	07:40–08:28	08:35	08:13–08:58	0.059	0.912
Sleep duration^b^	07:03	06:34–07:29	07:39	07:15–08:01	0.051	0.915
Habitual objectively measured sleep–wake timing (actigraphy), hh:mm						
Bedtime^a^	23:24	22:59–23:49	23:16	22:54–23:39	0.668	0.152
Get‐up time^a^	07:28	07:04–07:51	07:29	07:09–07:50	0.910	0.200
Time in bed^b^	07:55	07:37–08:13	08:17	08:01–08:33	0.072	0.832
Self‐reported sleep quality						
PSQI, range = 0–21^d^	4.1	3.1–5.3	4.4	3.4–5.6	0.688	0.935
FOSQ, range = 5–20^d^	18.1	16.9–19.3	17.5	16.4–18.6	0.439	0.586
Sleepiness						
Time of evening tiredness (MEQ)^a^	22:35	22:12–22:58	22:12	21:52–22:32	0.145	0.013^c^
Subjective sleepiness (ESS), range = 0–24^d^	5.6	4.0–7.1	7.1	5.7–8.6	0.149	0.292

Estimates (least squares means) and 95% CIs are indicated for each group adjusted for age and sex. For each measure, the group effect (controls vs Pre‐HD) and the disease burden effect (DBS) are indicated. Group analysis involved a mixed model analysis of variance controlled for age and sex. Effects of age and sex are not indicated. Effect of DBS is analyzed within the Pre‐HD group using a multivariate regression including DBS and sex. Effect size is indicated only for significant effects.

a Clock time.

b Hours:minutes.

c Significant effects.

d Total score.

CI = confidence interval; DBS = disease burden score; ESS = Epworth Sleepiness Scale; FOSQ = Functional Outcomes of Sleep Questionnaire; MEQ = Morningness‐Eveningness Questionnaire; Pre‐HD = premanifest Huntington disease; PSQI = Pittsburgh Sleep Quality Index.

**Table 5:** Group Differences in the Studied Metabolic Parameters

	Controls			Pre‐HD				
Studied Measures	No.	Estimate	95% CI	No.	Estimate	95% CI	Group Main Effects, *p* (Cohen *f* 2	DBS Effect, *p*
Body composition, DEXA scan^a^								
Fat‐Z	31	0.9	0.4 to 1.4	31	1.0	0.6 to 1.5	0.714	0.5647
Lean‐Z	31	−0.1	−0.5 to 0.2	31	−0.1	−0.4 to 0.2	0.883	0.8342
Mass, kg	31	74.1	69.4 to 79.1	31	76.0	71.6 to 80.8	0.591	0.4324
BMD‐Z	31	1.0	0.6 to 1.3	31	0.5	0.2 to 0.9	0.102	0.5273
Hormonal serum biochemistry								
Leptin, ng/ml^b^	24	10.2	8.4 to 12.3	27	10.1	8.5 to 11.8	0.919	0.1382
Testosterone, nmol/l^a^	24	9.9	8.1 to 11.8	25	8.8	7.2 to 10.5	0.373	0.2954
Cortisol, nmol/l^a^	23	375.3	330.9 to 425.7	26	347.3	312.6 to 386.0	0.346	0.7201
Vitamin D3, nmol/l^a^	24	55.0	43.3 to 69.7	26	56.1	45.6 to 68.9	0.899	0.0618
Energy expenditure								
36‐hour IC								
BMR, kJ/day^c^	31	6,575.8	6,331.2 to 6,830.5	31	6,722.1	6,491.5 to 6,960.8	0.374	0.0861
SMR, kJ/day^c^	31	6,060.1	5,859.8 to 6,260.3	31	6,082.2	5,898.0 to 6,266.4	0.865	0.5038
TEE, kJ/day^d^	30	9,652.3	9,359.1 to 9,945.6	30	9,394.2	9,123.6 to 9,664.7	0.202	0.3259
AEE, kJ/day^d^	30	2,923.2	2,716.4 to 3,099.8	30	2,554.2	2,278.2 to 2,772.8	0.018 (0.12)^e^	0.4648
Energy balance, kJ/day^d^	29	−161.8	−808.1 to 484.5	27	29.2	−585.6 to 644.0	0.669	0.6626
DLW								
TEE, kJ/day^d^	36	11,856.3	11,352.7 to 12,382.2	37	11,252.1	10,763.4 to 11,763.0	0.098	0.3617
AEE, kJ/day^d^	24	5,224.4	4,663.8 to 5,785.1	31	4,641.1	4,131.8 to 5,150.4	0.129	0.0887
Combined IC‐DLW								
PAL (TEE/BMR)^d^	24	1.8	1.7 to 1.8	31	1.7	1.6 to 1.7	0.106	0.1297
VPA, kJ/day^d^	24	2,476.9	1,876.7 to 3,077.1	31	2,219.9	1,667.6 to 2,772.1	0.531	0.05

Number of participants in a category, estimates (least squares means), and 95% CIs are indicated for each group adjusted for multiple covariates. The energy intake during the indirect calorimetry was normalized for each participant's need, based on the BMR predicted using Schofield's formula.^46^ For each measure, the group effect (controls vs Pre‐HD) and the disease burden effect (DBS) are indicated. Group analysis involved a mixed model analysis of variance controlled for age, sex, and further covariates as described above. Effects of covariates are not indicated. Effect of DBS is analyzed within the Pre‐HD group using a multivariate regression including the same covariates as for the group analyses except age. Effect size is indicated only for significant effects.

a Estimates controlled for age and sex.

b Estimates controlled for age, sex, and fat mass.

c Estimates controlled for age, sex, and lean mass

d Estimates controlled for age, sex, and total mass.

e Significant effects.

AEE = activity energy expenditure; BMD‐Z = bone mineral density normalized by age and sex; BMR = basal metabolic rate; CI = confidence interval; DBS = disease burden score; DEXA = dual‐energy x‐ray absorptiometry; DLW = doubly labeled water; Fat‐Z = fat normalized by age and sex; IC = indirect calorimetry; Lean‐Z = lean mass normalized by age and sex; PAL = physical activity level (TEE from DLW study/BMR from 36‐hour IC study); Pre‐HD = premanifest Huntington disease; SMR = sleep metabolic rate (overnight); TEE = total energy expenditure; VPA = variance of physical activity (TEE from DLW study − TEE from 36‐hour IC study; see Subjects and Methods).

The first hypothesis tested was whether the premanifest patients differed from controls. This was examined by using PC1 (or PC2 for the metabolic data) as the outcome variable in a linear model with age, sex, and group (control vs Pre‐HD) as predictor variables. The sleep study had an additional group of early manifest HD patients who were added to the analysis for comparative purposes (positive control). Pairwise differences between the control, Pre‐HD, and early HD groups were examined with Tukey honestly significant difference (HSD) post hoc test. The second hypothesis examined whether abnormalities were associated with DBS. This was tested by using the PCs as the outcome variable in a linear model with sex and DBS as predictor variables (age was not included as it would partial out age from the disease burden effect). We then based our conclusions about the presence of abnormalities on the results of the PCA, which involved only 2 tests for each of the 4 domains (8 statistical tests in total, with the post hoc tests for the sleep study corrected for multiple testing). We then undertook the further step of controlling for the “manuscriptwise” false‐positive rate by adjusting the 8 key *p*‐values for multiple testing across the 4 domains using the Holm–Bonferroni method.

One difficulty with the use of the PCA approach is interpreting what the PCs represent in terms of the original variables. However, we avoided this by examining the loadings of the variables that have contributed the most to any PC. The loadings are therefore the correlation between the original variables and the PC and help interpret the underlying construct or latent feature that is measured by these original variables. The main variables contributing to each of the PCs are reported with their loadings in the results section and are shown in the figures, and we also present the results for the individual variables in Tables 2 to 5. The tables contain secondary exploratory analyses that we have not corrected for multiple testing; as such, some of the individual variables may therefore be false positives, but inference about abnormalities in premanifest patients and the strong control for the false‐positive rate are done at the latent variable level. The results of the individual variables are thus more descriptive than inferential and are presented as a reference for other published and future studies.

The relative power spectrum of EEG activity was analyzed separately for the whole night REM and NREM periods. The same 2 statistical models described above were used but the outcome was a 1Hz spectral bin rather than a principal component. A separate model was fitted for each bin from 1 to 40Hz, using either disease group as the main predictor variable and adjusting for age and sex, or DBS as the main predictor variable and adjusting for sex. To minimize the number of false positives, we applied a Bonferroni correction (α = 0.05/40 = 0.00125). This is a conservative correction, given that the EEG power was highly correlated (the median correlation between adjacent frequency bins was 0.99, and the Lag 1 autocorrelation within patients was also >0.99.) We also report the original uncorrected results to avoid false negatives.

Measures of energy expenditure were adjusted for lean mass and leptin was adjusted for fat mass. The energy intake was normalized for each participant's need, based on the BMR predicted using the Schofield formula.^46^ The magnitude of the significant statistical effects was marked by Cohen's *f* 47:

$$f^{2}=u/v\times F$$

where *u* and *v* are, respectively, the numerator and denominator degrees of freedom of the *F* statistic used to determine the main effect in the analysis. Analyses were performed with SAS (version 9.2) and R (version 3.1.3).

## Results

**Figure 1:**
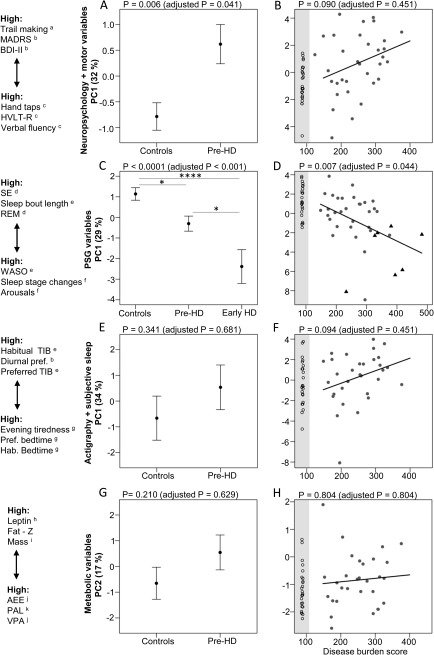
Principal components (PCs) by Group (A,C,E,G) and by disease burden (B,D,F,H) for each of the studied domains. The y‐axis in each graph is a PC, and the amount of variation it accounts for is indicated in parentheses. The original variables contributing to each component are shown on the far left, ranked according to their PC loading, which aids interpretation. For example, premanifest (Pre‐HD) patients have high values on PC1 for the neuropsychological and motor performance variables (A). These correspond to high values on the Trail Making Tests A and B, the Montgomery–Åsberg Depression Rating Scale (MADRS), and the Beck Depression Inventory II (BDI‐II), which indicate worse executive and psychomotor performance and elevated depression. Similarly, the controls have low values on PC1, and this corresponds to high values on the Verbal Fluency Test, Hopkins Verbal Learning Test–Revised (HVLT‐R), and Hand Tapping Test, which indicate better executive, verbal memory, and motor performance. The variables are ranked according to their loadings; only the top 3 are indicated; variables at the ends of the y‐axis have higher absolute loadings. For the individual PC loadings, please refer to the results section. The graphs on the left (A, C, E, G) show group differences between the PCs. Estimates (least squares means) and standard error of the mean are indicated, controlled for age and sex. The graphs on the right (B, D, F, H) show the association between the PCs and disease burden score controlled for sex within the gene carrier group; controls *(black open circles)* are shown for reference *(shaded area)* but are not included in the analysis. Grey filled circles = Pre‐HD; black triangles = early Huntington disease (HD). Original and adjusted *P*‐values are shown in parentheses and these are adjusted for all 8 tests shown in the figure using the Holm–Bonferroni method. As the polysomnography (PSG) data include a small group of early manifest HD (Early HD), *p*‐values indicate the main effect of group, and pairwise comparisons show that each group is different from every other group (Tukey honestly significant difference post hoc test: *p < 0.05, ****p < 0.0001). Results of the individual variables can be found in Tables 2 to 5. For detailed description of the variables, please refer to Subjects and Methods and Tables 2 to 5. ^a^seconds, ^b^total score, ^c^total correct, ^d^percentage, ^e^minutes, ^f^number per hours of sleep, ^g^clock time, ^h^ng/ml, ^i^kg, ^j^kJ/day; ^k^ratio. AEE = average daily activity–related energy expenditure; Hab. = habitual; PAL = physical activity level; Pref. = preferred; REM = rapid eye movement sleep; SE = sleep efficiency; TIB = time in bed; VPA = variance of physical activity; WASO = wake time between sleep onset and final awakening.

The cognitive, psychiatric, and motor profile of the studied premanifest group reproduced the previously reported abnormalities in Pre‐HD.^48^ PCA showed that the first component (PC1) explained 32% of the variation across all measures and was significantly different between the groups (*p* = 0.006, adjusted *p* = 0.041), indicating that Pre‐HD participants had poorer performance independent of age and sex (Fig 1A). Premanifest patients with higher DBS scores tended to have higher values on PC1, but the association was not significant (*p* = 0.090, adjusted *p* = 0.451; see Fig 1B).

PC1 included performance at Trail Making B (PC loading = 0.69), Trail Making A (PC loading = 0.54), total scores on the MADRS (PC loading = 0.47), BDI‐II (PC loading = 0.36), phonemic fluency (PC loading = −0.76), verbal memory (PC loading = −0.75), semantic fluency (PC loading = −0.66), left hand taps (PC loading = −0.61), MoCA (PC loading = −0.59), right hand taps (PC loading = −0.59), olfactory identification (PC loading = 0.56), and olfactory discrimination (PC loading = −0.38). The higher positive and negative PC loading values indicate higher positive or negative correlations with PC1. As the groups were clearly differentiated along the PC1 axis, the positive PC loading values indicate higher scores in Pre‐HD than controls, whereas the negative values indicate higher scores in controls than Pre‐HD.

The secondary exploratory and noninferential analyses showed that the global cognitive performance (MoCA), verbal memory (HVLT‐R), and left hand tapping scores discriminated the studied groups independently of age and sex. MoCA score, olfactory discrimination, and verbal memory were associated with disease burden independent of sex (see Table 2).

### Laboratory Measured Sleep Quality and Sleepiness

For the PSG‐measured sleep parameters, we also included a small group of early manifest HD patients (early HD) studied previously^10^ for comparative purposes. The PC1 component explained 29% of the variation across all PSG‐studied sleep measures (see Table 3), and all participants, and was significantly different across the 3 groups (*p* < 0.0001, adjusted *p* = 0.001), showing that the controls had a significantly better overall sleep quality compared to premanifest (Tukey HSD post hoc: *p* = 0.017) and early HD participants (Tukey HSD post hoc: *p* < 0.0001) independent of age and sex (see Fig 1C). Early manifest patients had a significantly worse sleep quality as compared to premanifest patients as well (Tukey HSD post hoc: *p* = 0.02). Importantly, the PC1 component was significantly associated with DBS in the entire premanifest and manifest gene carrier group (*p* = 0.0074, adjusted *p* = 0.044), presenting a linear drop in sleep quality with increasing disease burden independent of sex (see Fig 1D). PC1 included (for a detailed description of the variables see Table 3): sleep efficiency (PC loading = 0.76), length of continuous sleep bouts (PC loading = 0.70), REM sleep (percentage of total sleep time [TST]; PC loading = 0.65), TST (PC loading = 0.55), length of continuous REM bouts (PC loading = 0.39), number of arousals (shifts to stage 1 sleep, wake, or movement per hour of sleep; PC loading = −0.94), total number of shifts between sleep stages per hour of sleep (PC loading = −0.86), wake time between sleep onset and final awakening (WASO; PC loading = −0.86), number of fast shifts between sleep stages (periods of 1.5 minutes with 3 different 30‐second‐long sleep stages per hour of sleep; PC loading = −0.85), stage1 sleep (percentage of TST; PC loading = −0.75), latency to persistent sleep (10 minutes of continuous sleep; PC loading = −0.59), apnea–hypopnea index (apnea and hypopnea events per hour of sleep; PC loading = −0.53), latency to slow wave sleep (SWS; PC loading = −0.47), and latency to REM sleep (PC loading = −0.38).

As the groups were clearly segregated along the PC1 axis, the positive PC loading values indicate higher score in controls than in premanifest gene carriers and manifest patients, and the negative values indicate higher scores in gene carriers than in controls. Importantly, the variables top ranked according to their PC loading value were measures of sleep continuity, indicating that this is the dimension of sleep quality that best correlates with PC1 and so contributes most to the differentiation of the studied groups along the PC1 axis.

**Figure 2:**
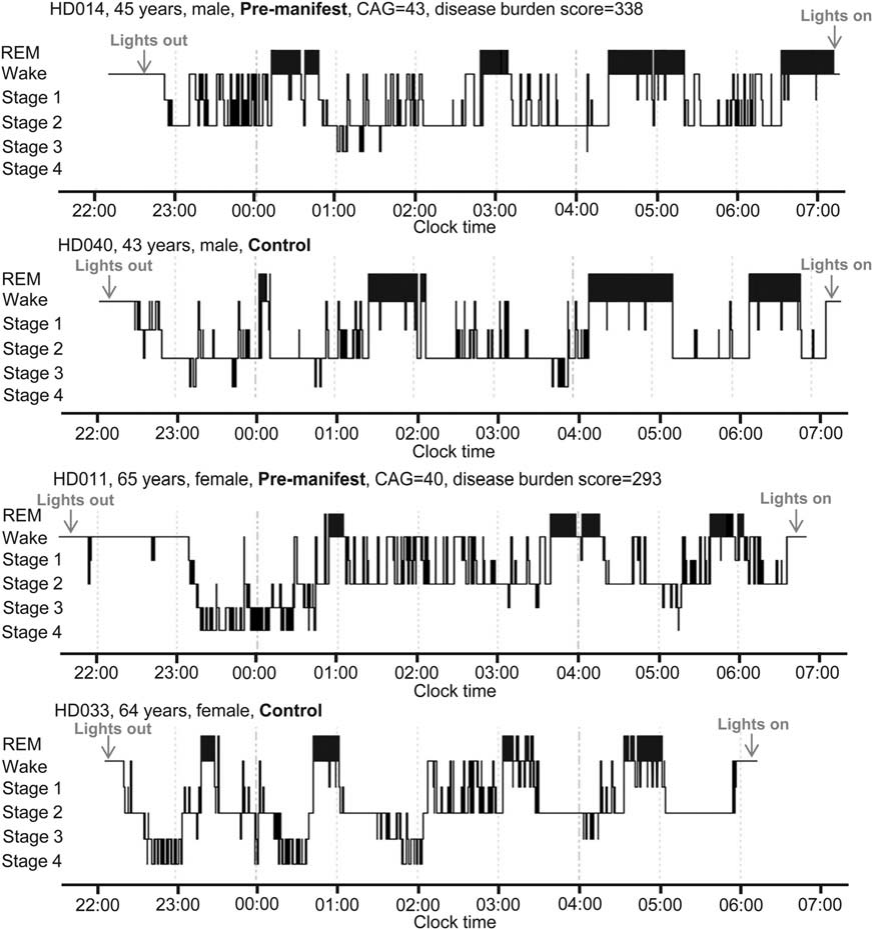
Representative sleep profiles of 2 premanifest participants and 2 age‐ and sex‐matched controls showing a more fragmented sleep profile in the premanifest participants. CAG = cytosine–adenosine–guanosine repeats; REM = rapid eye movement sleep.

The secondary exploratory analyses revealed that the 3 groups were different across a large range of sleep quality measures, indicating progressively worsening sleep phenotype from premanifest to manifest stage independent of age and sex (see Table 3). This was characterized by an earlier bedtime, longer time in bed, lower sleep efficiency, increased stage 1 sleep, decreased REM sleep, and a more fragmented sleep profile across several measures of sleep continuity. Pre‐HD participants presented longer WASO, increased sleep fragmentation (Fig 2) across multiple measures, and higher daytime sleepiness compared to controls (see Table 3). Pre‐HD participants were sleepier than both controls and early HD participants, as indicated by a shorter average sleep latency measured by MSLT. The early HD group presented the smallest sleep propensity during the day independent of age and sex. Daytime sleepiness decreased with disease burden in the premanifest and manifest gene carrier group.

### Sleep–Wake Timing and Self‐Reported Measures of Sleep

As sleep–wake timing and diurnal preference have been associated with individual differences in sleep quality,^49^ metabolism,^50^ and mental health,^51^ we also assessed this aspect of sleep. PC1 component explained 34% of the variation across all studied measures within this domain (see Table 4). It was not different between the groups (*p* = 0.341, adjusted *p* = 0.681; see Fig 1E) and was not associated with DBS (*p* = 0.0945, adjusted *p* = 0.451; see Fig 1F), although the direction of the effects was consistent with the objective PSG assessments of sleep quality. PC1 included actigraphy‐measured habitual time in bed (TIB; PC loading = 0.60), diurnal preference measured by the MEQ total score (PC loading = 0.60), preferred TIB (PC loading = 0.56), self‐reported habitual TIB (PC loading = 0.44), sleep duration (PC loading = 0.43), and bedtime (PC loading = −0.92), actigraphy measured (PC loading = −0.90) and preferred bedtime (PC loading = −0.89), time of self‐reported evening tiredness sufficient to go to sleep (PC loading = −0.87), actigraphy measured (PC loading = −0.57) and self‐reported habitual getup time (PC loading = −0.51), and preferred getup time (PC loading = −0.38).

The secondary exploratory analyses did not show group differences across any of the measures. There was an association between an earlier timing of evening sleepiness sufficient to go to sleep and increasing disease burden independent of sex (see Table 4). We consider that reporting these values is important, as in particular chronotype and diurnal preference are important characteristics of the studied population and allow for systematic comparison of different study groups.

### EEG Spectral Characteristics

**Figure 3:**
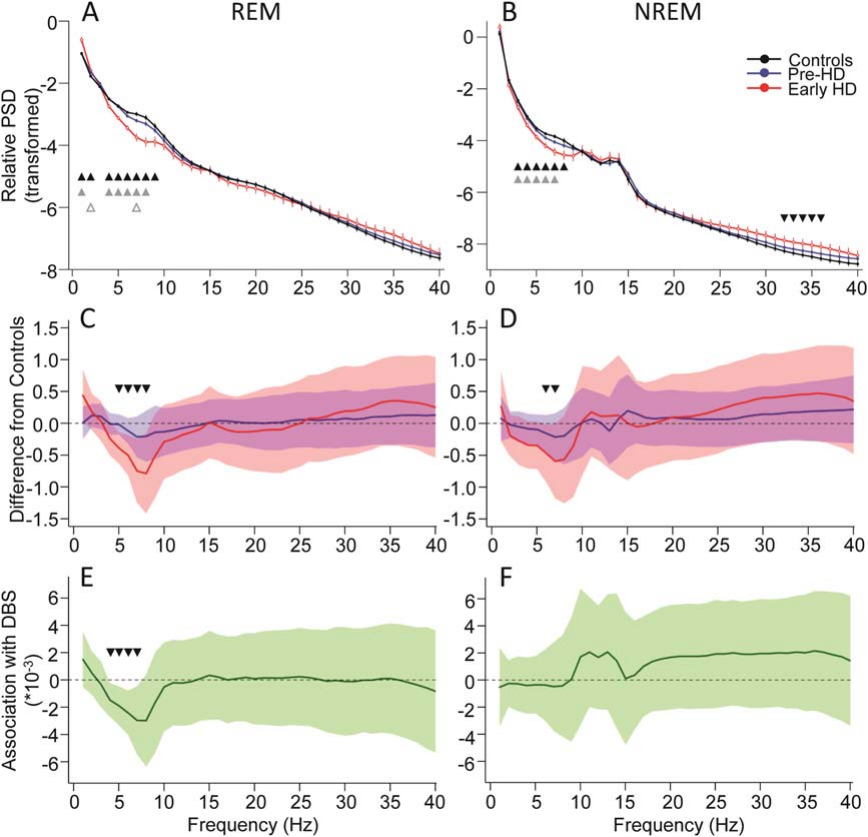
Characteristics of the relative electroencephalographic power spectral density (PSD) measured during rapid eye movement (REM; A, C, E) and non‐REM (NREM; B, D, F) sleep in controls, premanifest (Pre‐HD) patients, and early manifest Huntington disease (HD; Early HD) patients. (A, B) The average transformed (*y* = log[*x*/(1−*x*)] see Subjects and Methods) relative power spectrum density measured during REM (A) and NREM (B) sleep for each group is presented as estimates (least squares means) and standard error of the mean controlled for age and sex. Triangles indicate significant (*p* < 0.05) group differences (2‐tailed *t* test). Black triangles = controls versus Early HD, gray solid triangles = Pre‐HD versus Early HD, gray open triangles = controls versus Pre‐HD. (C, D) Mean difference from controls in the averaged relative power spectrum during REM (C) and NREM (D) sleep is presented with 99.875% confidence interval (CI) limits controlled for age and sex. Dashed reference line = Control; blue lines = Pre‐HD; red lines = Early HD. Triangles indicate statistically corrected significant (*p* < 0.00125) differences between Early HD and controls. (E, F) Association between the relative power spectrum measured during REM (E) and NREM (F) sleep and the disease burden score (DBS) within the entire premanifest and manifest gene carrier group. Regression coefficients and 99.875% CI are indicated. Triangles indicate statistically corrected significant associations (*p* < 0.00125) with DBS.

The whole night REM sleep–dependent relative EEG power showed a major decrease in the frequency range of 4 to 9Hz and an increase at 1 to 2Hz in early HD as compared to controls independent of age and sex (Fig 3). The pre‐HD group was mostly intermediate between the 2 groups or similar to controls, except for the 2Hz and 7Hz bins, where they were similar to early manifest patients showing a higher (2Hz) and lower (7Hz) relative power, respectively, as compared to controls. After conservative statistical correction, only the relative power differences within the 5 to 7Hz frequency range remained significant between the early HD and the control group. The REM sleep–dependent 4 to 7Hz range showed a robust association with disease burden independent of sex, and this survived conservative statistical correction. In NREM sleep, there was a similar decrease in the relative power in early HD compared to controls and Pre‐HD in the range of 3 to 8Hz, but also an increase in the 32 to 36Hz high‐frequency range compared to controls independent of age and sex. After conservative statistical correction, only the decrease in the 6 to 7Hz range remained significant without any significant association with disease burden.

### Metabolic Alterations

For the metabolic analyses, we controlled our analyses for age, sex, and body mass, fat mass, or lean mass depending on the studied measures (see Table 5). As PC1 was dominated by sex effects, we analyzed PC2, which explained 17% of the variation across all studied metabolic measures and showed no group effect (adjusted *p* = 0.21; see Fig 1G) nor association with DBS (*p* = 0.804; see Fig 1H). PC2 included leptin (PC loading = 0.70), fat mass *Z* score (PC loading = 0.61), total body mass (PC loading = 0.50), VPA (PC loading = −0.61), PAL (PC loading = −0.55), AEE (PC loading = −0.52), and TEE (PC loading = −0.37) measured by DLW and testosterone (PC loading = −0.37).

Secondary exploratory analyses showed no significant group differences except a decreased AEE in premanifest patients as compared to controls independent of age, sex, and body mass (see Table 5).

## Discussion

Sleep is of upmost importance in life, with multiple links to cognition^52^ and metabolism.^53^ In manifest HD, disturbances have been reported in all 3 of these areas, but have not been systematically studied in patients prior to motor disease onset. The findings reported here represent the first comprehensive study of sleep and metabolism in a group of Pre‐HD individuals.

We show that objectively measured sleep quality is greatly affected ahead of overt disease onset including various intercorrelated sleep parameters. That sleep continuity measures such as number of arousals, total number of shifts between sleep stages, wake time after sleep onset, and fast changes in sleep stages had the highest contribution (PC loading) to PC1 indicates that sleep continuity is the major sleep characteristic differentiating the studied groups and is significantly associated with disease burden. This interpretation is supported by the secondary exploratory analyses. Whereas the TST and the relative duration of sleep stages are unchanged, the continuity of sleep is decreased in premanifest patients. Sleep continuity is thought to be important for the restorative function of sleep.^54^ This suggests that even in the presence of sufficient sleep duration, the restorative effects may be compromised during the premanifest stage. These results of fragmented sleep are in line with a recent paper showing major sleep disturbances in manifest HD patients that were also characterized by increased arousals and awakenings^55^ and data from transgenic animal models of HD showing that sleep quality as well as oscillatory brain activity (EEG) and circadian rhythmicity becomes gradually more disrupted as the disease progresses.^11^, ^12^, ^14^ Furthermore, it has recently been shown that resting EEG alterations in pre‐HD individuals may be related to the course of the pathological process and to HD endophenotype.^43^ These results are intriguing, because EEG activity reflects characteristics of cortical and subcortical neural activity and has the potential to become a biomarker for HD onset and progression in the future.^56^ Whereas there are a number of quantitative EEG (qEEG) studies of waking brain activity in HD (for a review see Nguyen et al^56^), there are only a couple of studies looking at spectral features of EEG during sleep, and only in transgenic animal models of HD,^11^, ^12^, ^14^, ^57^, ^58^ with no studies in humans. Our study therefore is the first qEEG study performed during sleep in premanifest and manifest patients with HD and indicates alterations in the NREM‐ and particularly the REM‐dependent oscillatory activity of the brain associated with disease burden in gene carriers. Some of our findings such as the decrease in the 3 to 8Hz range and the increase in the high‐frequency (32–36Hz) range in the early HD group are somewhat similar to findings in transgenic animal studies reporting a progressive decrease in low‐frequency (delta) and increase in high‐frequency (beta–gamma) activity in sleep.^11^, ^12^, ^58^ The REM‐dependent increase in the 1 to 2Hz range is unexpected and could be related to increased REM intensity (density of REM), a hypothesis that remains to be tested. However, all these effects were small, affecting only the early HD group, with a marginal association with the disease burden at best. Therefore, a detailed discussion on this is not justified. It is important however to emphasize that we did not analyze EEG segments where increased muscle activity contaminated the EEG signal. Therefore, sleep EEG segments around awakenings when elevated muscle activity was usually present were excluded from the spectral analyses, which may have masked to a degree the increase in the high‐frequency EEG activity in the gene carrier group presenting with more frequent awakenings.

The most robust qEEG finding, however, that survived conservative statistical correction is a decrease in the 4 to 8Hz frequency band in REM and in the 6 to 7Hz range in NREM sleep in the early HD group independently of age and sex, pointing to a theta frequency range–specific alteration in HD. Although premanifest patients were not significantly different from controls, importantly the REM‐dependent theta (4–7Hz) decrease was significantly associated with disease burden score in the entire gene carrier group. Theta activity during NREM and REM sleep has been shown to have a similar age‐dependent decline, indicating probably common functional correlates, and it has been suggested to play a role in neural restoration following wakefulness.^59^ This is supported by the finding that sleep deprivation increases EEG activity in the 1 to 7Hz frequency range during recovery sleep in both NREM and REM sleep episodes.^60^ The functional correlates of this specific EEG pattern and the possible underlying mechanisms in HD remain to be established.

Now altered sleep continuity in ageing is thought to be at least in part a consequence of a “weakened” circadian signal,^61^ and in HD it is known that melatonin levels are already reduced at a premanifest stage.^62^ This coupled with the unstable sleep phenotype that we describe in this paper suggests a deficit in neural processes regulating vigilance stages. Sleep and wakefulness result from interacting neurotransmitter systems in the brainstem, hypothalamus, and basal forebrain,^63^ with sleep emerging from the inhibition of wake‐promoting systems by the preoptic area of the hypothalamus.^64^ Therefore, it may be that one of the earliest sites of pathology in HD is the hypothalamus.^21^

The results also show that sleep disturbances similar to the cognitive deficits follow a linear association with disease burden independent of sex. Although both the primary cognitive and sleep deficits appear long before the overt motor disease onset, the mechanistic link between these factors remains to be established. The sleep data show that once the disease becomes manifest, many other sleep problems emerge such as a decrease in sleep efficiency and REM sleep and an increase in superficial stage 1 sleep, which are in line with previous studies performed in manifest patients.^55^, ^65^ We also show that these effects are independent of age and sex.

Our exploratory analyses show that objectively measured daytime sleepiness is greater in premanifest patients than controls, and then decreases with increasing disease burden and is much less in early manifest patients as compared to both controls and premanifest individuals, independent of age and sex. This is an intriguing finding, as one would expect the greater sleep deficits to lead to greater sleep propensity. Healthy aging is associated with a reduction in daytime sleep propensity, sleep continuity, and SWS and may reflect a lessening in homeostatic sleep requirement,^66^ which is related to synaptic strength and plasticity.^67^ Therefore, there may be a complex mechanistic link between the progressive decrease in daytime sleepiness and increasing sleep deficits in HD involving a gradually worsening synaptic pathology, which may also account for some of the early cognitive deficits. It is also known that patients entering the early manifest stages of the illness lose a degree of insight.^68^ Although we did not explicitly look at this in this study, it may help explain the discrepancy between the subjective and objective sleep abnormalities reported previously in early manifest HD^10^ and now in premanifest patients. This further raises questions on the usefulness of employing subjective measures of sleep quality in HD clinical practice and research.

In addition, our metabolic study (which is the first to investigate energy expenditure in Pre‐HD patients using both the field and laboratory environments) did not find any metabolic abnormality at this stage of the condition.^20^

Although our study has many unique qualities, it also has a number of limitations. Although this is so far the only study to systematically investigate sleep, cognition, and metabolism in a cohort of premanifest patients using state of the art methodology, the results presented here are cross‐sectional, and so we are not able to establish any causality between abnormalities. We are therefore continuing to follow up these patients to evaluate how the abnormalities identified change as the patient transitions into manifest disease states. Furthermore, not all participants took part in all studies, which were not all performed at the same time in some participants. However, the sleep components of the work preceded all the other studies in all cases, indicating that any time difference between the studies could not cause the temporal primacy of the sleep abnormalities. Additionally, some of our patients and controls were taking medications that may affect sleep and metabolism, but importantly the groups were not significantly different in this regard and our most important results (such as sleep fragmentation) remained highly significant even after excluding the medicated participants.

In summary, we have shown for the first time that the premanifest stage of HD is characterized by sleep abnormalities at a time when the well‐described early cognitive disturbances begin to emerge. Metabolic abnormalities related to body composition and energy expenditure are not present at this stage of the disease, suggesting that sleep may be one of the earliest homeostatic processes to go wrong in HD, which in turn may have other effects such as driving early cognitive dysfunction and even the pathology of HD itself.

## Authorship

R.A.B., A.O.G.G., J.M.S., P.R.M., and B.B. designed the study. A.S.L., F.P., and A.O.G.G. ran the study. A.S.L., A.O.G.G., F.P., and S.L.M. collected the cognitive and clinical data. J.M.S. and L.R. collected the sleep data. P.R.M. and L.P.E.W. performed the indirect calorimetry and body composition assessment. P.S. prepared the DLW and processed the urine samples. A.S.L., Z.I.L., and S.E.L. analyzed the sleep EEG data. A.S.L., F.P., A.O.G.G., and S.E.L. managed and analyzed all other data. A.S.L., F.P., S.E.L., S.L.M., B.B., Z.I.L., and R.A.B. interpreted the data and wrote the paper. All authors were involved in the revision of the manuscript. A.S.L. and F.P. are equal first authors.

## Potential Conflicts of Interest

Nothing to report.
